# Study on the correlation between leisure activities and cognitive impairment in middle‐aged and elderly people in High‐altitude environment

**DOI:** 10.1002/alz.087914

**Published:** 2025-01-03

**Authors:** Aiqin Zhu, Changbiao Chai

**Affiliations:** ^1^ Institute of Geriatric, Qinghai Provincial Hospital, Xining China; ^2^ Clinical Research Center for Geriatric Diseases of Qinghai, Xining China; ^3^ Graduate School of Qinghai University, xining China; ^4^ Qinghai Provincial People’s Hospital, xining China

## Abstract

**Background:**

To explore the influence of leisure activities on cognitive function of middle‐aged and elderly people living in hypoxia environment.

**Method:**

Using a cross‐sectional random sampling survey method, a total of people over 50 years old who have lived for more than 20 years (average altitude 3000m) in Qinghai plateau region were selected. Demography information, chronic medical history, economic and marital status, and 21 leisure activities (including entertainment, mobile phone use, games, sports, travel, social interaction and housework) were investigated. Cognitive domain assessment tool was from Beijing aging brain rejuvenation initiative (BABRI) Dementia Risk Assessment program.

**Result:**

Among the 500 cases studied, there were 193 cases of cognitive impairment (CI) (38.6%), including 110 (22%) mild cognitive impairment and 83 (16.6%) Alzheimer’s disease. The CI group showed a significant increase in women, older age, workers and farmers, lower education, divorce and widowhood, living alone/with children, lower monthly income, smokers and drinkers (P<0.05); while reading (newspapers, magazines and books), playing chess and card activities (chess, poker and mahjong), watching TV, using mobile phones (Tiktok, Kwai and chatting), performing aerobic (walking, jogging, cycling and swimming) and resistance exercise (dumbbells, sandbags and pullers), traveling, visiting friends /relatives significantly decreased compared with the normal cognitive group (P<0.05). Multivariate binary regression analysis showed that women (OR = 1.787, 95% CI = 1.137‐2.808, P = 0.012), advanced age (OR = 1.066, 95% CI = 1.040‐1.093, P<0.001), occupation as workers (OR = 2.640, 95% CI = 1.263‐5.519, P = 0.010), and farming (OR = 2.420, 95% CI = 1.351‐4.333, P = 0.003) were risk factors for CI. Living with a spouse (OR = 0.211, 95% CI = 0.081‐0.548, P<0.001) and stop drinking (OR = 0.275, 95% CI = 0.093‐0.813, P = 0.020) were protective factors for CI. In leisure activities, aerobic endurance exercise (OR = 0.811, 95%CI = 0.676 ‐0.973, P = 0.024) and resistance exercise (OR = 0.625, 95%CI = 0.442 ‐0.883, P = 0.008) were protective factors for CI, while mobile phone use (OR = 1.185, 95% CI = 1.003‐1.339, P = 0.046) is a risk factor for CI. Mobile phone use (OR = 1.185, 95% CI = 1.003‐1.339, P = 0.046) was a risk factor for CI.

**Conclusion:**

Aerobic and resistance exercise have a protective effect on cognitive function, while long‐term mobile phone watching Tiktok, Kwai and chatting may lead to CI in middle‐aged and elderly people in hypoxia environment.

